# Diversity, geographical distribution, and prevalence of *Entamoeba* spp. in Brazil: a systematic review and meta-analysis

**DOI:** 10.1051/parasite/2021028

**Published:** 2021-03-30

**Authors:** Andernice dos Santos Zanetti, Antonio Francisco Malheiros, Tatiane Amorim de Matos, Carolina dos Santos, Paula Franciene Battaglini, Luciana Melhorança Moreira, Larissa Maria Scalon Lemos, Solange Kimie Ikeda Castrillon, Denise da Costa Boamorte Cortela, Eliane Ignotti, Omar Ariel Espinosa

**Affiliations:** 1 Post-Graduation Program in Environmental Science, Faculty of Agricultural and Biological Sciences, State University of Mato Grosso (UNEMAT) Tancredo Neves Ave., 1095 – Cavalhada II Caceres 78217-042 Mato Grosso Brazil; 2 Residency in Infectious Diseases, Júlio Miller University Hospital, Federal University of Mato Grosso Luis Philippe Pereira Leite St., Alvorada Cuiabá 78048-902 Mato Grosso Brazil; 3 Faculty of Agricultural and Biological Sciences, State University of Mato Grosso (UNEMAT) Tancredo Neves Ave., 1095 – Cavalhada II 78217-042 Caceres Mato Grosso Brazil; 4 Department of Nursing, Faculty of Health Sciences, State University of Mato Grosso (UNEMAT) Tancredo Neves Ave., 1095 – Cavalhada II Caceres 78217-042 Mato Grosso Brazil; 5 Department of Medicine, Faculty of Health Sciences, State University of Mato Grosso (UNEMAT) Tancredo Neves Ave., 1095 – Cavalhada II 78217-042 Caceres Mato Grosso Brazil; 6 Faculty Estacio of Pantanal (Estacio FAPAN) São Luís, 2522 St – Cidade Nova Caceres 78201-000 Mato Grosso Brazil

**Keywords:** Parasitic disease, Amebiasis, Diarrhea, Zoonoses, Protozoan

## Abstract

The genus *Entamoeba* includes a variety of widely distributed species adapted to live in the digestive tracts of humans and a large variety of animals of different classes. The objective of this study was to investigate the prevalence, distribution, and molecular epidemiology of *Entamoeba* spp. in different classes of hosts in Brazil. Studies that analyzed hosts from several classes, including humans and domestic, wild, or captive animals, were considered. The pooled prevalence of *Entamoeba* spp. was calculated using the random-effects model. A total of 166 studies on humans and 16 on animals were included. The prevalence of *Entamoeba* spp. in the Brazilian population was 22% (95% CI: 21–24). The state with the highest prevalence was Paraiba with 72%, followed by Federal District with 53%, and Rondonia with 50%. In immunocompromized patients, the prevalence was 18%, and cancer (36%) was the most prevalent cause of immunosuppression. The prevalence of *Entamoeba* spp. in animal hosts was 12% (95% CI: 7–17). Captive wild animals and domestic farm animals showed the highest prevalence, with 16% and 15%, respectively. The species found more often were *E. coli* (86.5%), *E. dispar* (7.9%), and *E. histolytica* (3.1%). In conclusion, a high prevalence (22%) of *Entamoeba* spp. was found in the Brazilian population, with a prevalence of up to 50% mainly in the northern, northeastern, and central-western regions. The pathogenic species *E. histolytica* is distributed in most Brazilian regions, with significant prevalence percentages. Among animals, unidentified *Entamoeba* species were most prevalent in mammals.

## Abstract (fr)

Le genre *Entamoeba* comprend une variété d’espèces largement distribuées, adaptées à vivre dans le tube digestif des humains et une grande variété d’animaux de différentes classes. L’objectif de cette étude était d’étudier la prévalence, la distribution et l’épidémiologie moléculaire d’*Entamoeba* spp. dans différentes classes d’hôtes au Brésil. Les études qui ont analysé les hôtes de plusieurs classes, y compris les humains et les animaux domestiques, sauvages ou captifs, ont été prises en compte. La prévalence combinée d’*Entamoeba* spp. a été calculée à l’aide du modèle à effets aléatoires. Au total, 166 études sur l’homme et 16 sur les animaux ont été incluses. La prévalence d’*Entamoeba* spp. dans la population brésilienne était de 22 % (IC à 95 % : 21–24). L’état avec la prévalence la plus élevée était Paraiba avec 72 %, suivi du District fédéral avec 53 % et Rondonia avec 50 %. Chez les patients immunodéprimés, la prévalence était de 18 % et le cancer (36 %) était la cause la plus fréquente d’immunosuppression. La prévalence d’*Entamoeba* spp. chez les hôtes animaux était de 12 % (IC à 95 % : 7–17). Les animaux sauvages en captivité et les animaux domestiques d’élevage ont affiché la prévalence la plus élevée, avec respectivement 16 % et 15 %. Les espèces trouvées le plus souvent étaient *E. coli* (86,5 %), *E. dispar* (7,9 %) et *E. histolytica* (3,1 %). En conclusion, une prévalence élevée (22 %) d’*Entamoeba* spp. a été trouvée dans la population brésilienne, allant jusqu’à 50 % principalement dans les régions du nord, du nord-est et du centre-ouest. L’espèce pathogène *E. histolytica* est répartie dans la plupart des régions du Brésil, avec des pourcentages de prévalence importants. Parmi les animaux, les espèces d’*Entamoeba* non identifiées étaient les plus répandues chez les mammifères.

## Introduction

The genus *Entamoeba* includes a variety of anaerobic, unicellular, and monoxenic protozoan species adapted to live as parasites or commensals in the digestive tracts of humans and a large variety of animals of different classes [5, 7, 64, 110, 112, 205, 206].

The main species of this genus that parasitize humans are *E. histolytica*, *E*. *dispar*, *E. moshkovskii*, *E. coli*, *E. polecki*, *E. bangladeshi*, and *E. hartmanni* [84, 124, 151, 174]. Morphologically, the species *E. histolytica*, *E. dispar*, and *E. moshkovskii* are considered identical, but only *E. histolytica* is the causative agent of amebiasis, a gastrointestinal disease that commonly occurs worldwide; amebiasis is considered endemic in tropical regions and is associated with inadequate socioeconomic and sanitary conditions [8, 166, 216]. *Entamoeba histolytica* shows several degrees of virulence and is capable of invading a wide variety of tissues in the host, including those of the colon and liver, and more rarely the lung, skin, urogenital tract, brain, and spleen. This invasive feature separates it from the other species [70]. It is estimated that amebiasis accounts for 55 500 all-age deaths and causes disability-adjusted life years at 2.237 million [211].

In contrast, *E. dispar* can cause focal intestinal lesions in laboratory animals [133]. However, in humans, it is considered a stable commensal with no virulent characteristics, producing an asymptomatic carrier state and being generally much more prevalent worldwide than *E. histolytica* [64, 124]. On the other hand, the idea that *E. dispar* is a simple commensal parasite is under discussion, and some authors discuss the importance of this species in damage of the intestine and liver [73].

Globally, the overall prevalence of *Entamoeba* spp. in humans is 3.5%. *Entamoeba histolytica* and *E. dispar* account for 81.7% of this global prevalence in documented infections. The comparison of prevalence by regions showed differences in prevalence between Australia (1.7%) and North America (21.6%) [64].

Regarding zoonotic potential, research on *E. histolytica*, *E. dispar*, *E. hartmanni*, *E. coli*, *E. moshkovskii*, and *E. polecki* is remarkably important because of previous reports on these species in both humans and different species of animals worldwide [76, 110, 152, 165, 206]. Furthermore, regarding pathogenic potential, some of these species can cause diarrhea and other symptomatic presentations in non-human primates [165].

The *Entamoeba* spp. have a variety of vertebrate hosts: *E. moshkovskii* is found in cattle, elephants, and reptiles [94, 110]; *E. coli* and *E. hartmanni* are found in non-human primates [26, 57, 113, 220]; and finally, some studies suggest that different subtypes of *E. polecki*, infect human, non-human primates, pigs and ostriches [41, 59, 76, 84, 112].

In Brazil, several studies based on microscopic examination have investigated the prevalence of amebiasis in different population groups, but discriminatory studies between species (using molecular methods) are relatively scarce and mainly address different animal hosts. Although there are data on the prevalence of *Entamoeba* spp. in some regions, there is no aggregate analysis of the prevalence and distribution of species of this protozoan by geographic area, sex, age group, and host type in Brazil. Therefore, the objective of this systematic review and meta-analysis was to determine the prevalence and distribution of different species of *Entamoeba* in several host classes in Brazil.

## Materials and methods

The protocol of this systematic review was registered in the International Prospective Register of Systematic Reviews (PROSPERO 2019: CRD42020167222) before its implementation. The protocol and final report were developed according to the Cochrane Handbook for Systematic Reviews of Interventions [105].

### The review question

What is the prevalence and geographical distribution of *Entamoeba* spp. in different host species in Brazil?

### Inclusion and exclusion criteria

This review included studies on various hosts (humans and domestic, wild, or captive animals) of different classes to determine the prevalence and genetic identification of *Entamoeba* spp. in Brazil through coprological analyses and molecular techniques.

Studies analyzing fecal samples of humans and domestic, wild, or captive animals that did not report percentages of samples positive for *Entamoeba* spp. were excluded.

### Types of studies

This review included cross-sectional epidemiological studies assessing the prevalence of *Entamoeba* spp. in humans and wild, captive, and domestic animals.

### Search strategy

An initial search limited to MEDLINE was conducted using MeSH index terms and related keywords. Subsequently, the words contained in the title, abstract, and index terms used to describe the articles were analyzed. A second search using all identified keywords and index terms was performed using all included databases. As a source of gray literature, a search was conducted in the reference lists of dissertations and theses that evaluated the prevalence of protozoan intestinal parasites. Because this search was limited to Brazil, it was limited to studies in the English, Spanish, and Portuguese languages. This search had no start date limitation but was completed in November 2020.

The studies were searched in the following databases: Spanish Bibliographic Index of Health Sciences (*IBECS*), Latin American and Caribbean Literature in Health Sciences (*LILACS*), Virtual Health Library (*BVS*), US National Library of Medicine bibliographic database (Medline), Elsevier database EMBASE, Cumulative Index to Nursing and Allied Health Literature (CINAHL), Web of Science, Cochrane Library, and National Institute of Health and Clinical Excellence (NICE). The MeSH index terms searched were *Entamoeba* and Brazil. The keywords *Brasil* and *Endamoeba* were also included in the search. The MeSH terms and keywords were combined via the boolean operators “AND” and/or “OR” to compose the search strings.

### Assessment of methodological quality

The articles selected for data retrieval were analyzed by two independent reviewers to evaluate the methodological validity of each text before inclusion in this review. The quality of the publications included was evaluated based on the Grading of Recommendations Assessment, Development, and Evaluation (GRADE) criteria. Studies received one point for not presenting a study design or execution limitations (risk of bias), inconsistency of results, indirectness of evidence, imprecision, and publication bias. A score of 4–5 points was considered high quality, 3 as moderate quality, and 0–2 as low quality.

### Data extraction

The selected texts were evaluated by two independent reviewers for validity before inclusion; discrepancies were resolved by an independent reviewer. The data were entered into the Review Manager (RevMan 5.3) [168] software for analysis. A data extraction table was used to evaluate the quality of demographic data, study location, sample size, number of cases, number of positive cases, and diagnostic test.

### Data summary

The random-effects meta-analysis model was used to analyze the overall combined prevalence of *Entamoeba* spp. in humans and animals. The heterogeneity among studies was evaluated using I2-statistic, which shows the percentage of variation among studies. These analyses were performed using the Stata software, version 12.

## Results

**Figure 1:**
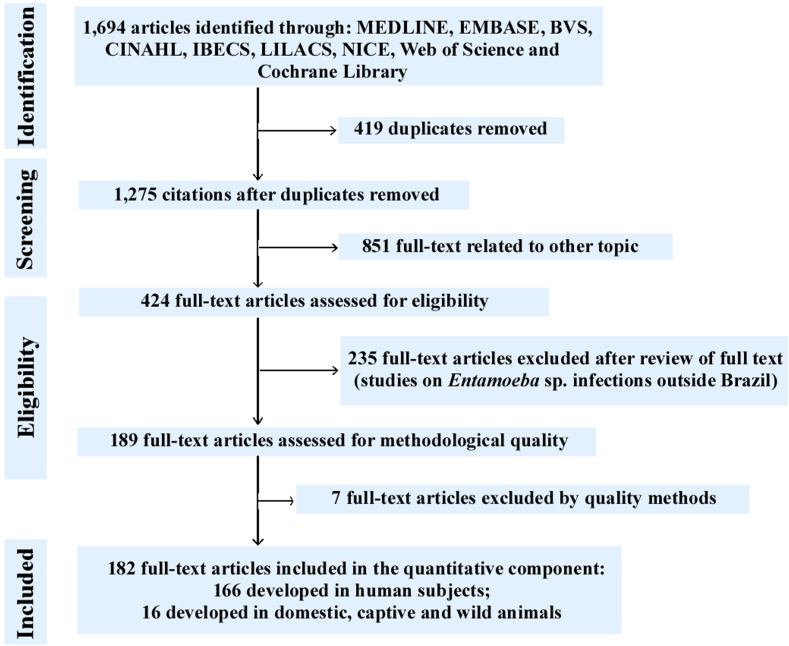
A flowchart of the steps performed in the systematic review.

Our systematic literature search yielded 1694 manuscripts using the established search strategies. As per the eligibility criteria (after exclusion of duplicate texts and articles related to other topics and exclusion of text based on review criteria or owing to method quality), 182 studies were selected for analysis (Table 1) [2–4, 6, 7, 9–25, 27–37, 39, 40, 42–45, 47–56, 58, 60–63, 65–69, 71, 72, 74, 75, 77–83, 85–93, 95–104, 106–109, 111, 114–123, 125–130, 132, 135, 136, 138–140, 142–146, 148–150, 153–164, 167, 169–173, 175–192, 194–204, 207–210, 212–215, 217–219]. Of these studies, 166 evaluated the prevalence of *Entamoeba* spp. in human fecal samples from different Brazilian states during different periods; the remaining 16 studies analyzed the prevalence of *Entamoeba* spp. parasites in different wild, captive, and domestic animals. Of the 182 studies included, 9 identified the species of the genus *Entamoeba* by molecular characterization, 17 by serology, and 2 by isoenzyme analysis. The results of this search strategy are presented in a Preferred Reporting Items for Systematic Reviews and Meta-Analyzes (PRISMA) flowchart (Fig. 1). Data were extracted according to the PRISMA Statement [141].

**Table 1:** A summary of the included studies.

No.	Region	City – State	Total *N*	Prevalence (%)	Diagnostic method	Author/year
Human host						
1	Midwest	Caceres – MT	53	9.4	C	Alencar et al. [7]
2	Midwest	Campo Novo do Parecis – MT	43	37.2	C	Zenazokenae et al. [219]
3	Midwest	Caceres – MT	183	36.6	C	Silva et al. [196]
4	Midwest	Rondonopolis – MT	215	11.5	C	Luz et al. [125]
5	Midwest	Parque do Xingu – MT	304	52.9	C	Escobar-Pardo et al. [77]
6	Midwest	MT	173	16.8	C	Coimbra Jr and Santos [60]
7	Midwest	Parque Xingu – MT	62	75.8	C	Ferreira et al. [87]
8	Midwest	Mirassol D’Oeste – MT	149	38.2	C	Latorraca et al. [118]
9	Midwest	Corumba – MS	200	52.0	C	Silva et al. [198]
10	Midwest	Corumba – MS	196	55.1	C	Silva et al. [197]
11	Midwest	Campo Grande – MS	510	4.6	C	Curval et al. [65]
12	Midwest	Campo Grande – MS	66	25.7	C	Higa Júnior et al. [104]
13	Midwest	MS	103	43.7	C	Neres-Norberg et al. [150]
14	Midwest	Bonito – MS	115	23.5	C	Gomes et al. [97]
15	Midwest	Sidrolandia – MS	313	64.8	C	Aguiar et al. [4]
16	Midwest	DF	75	53.3	C	Pereira et al. [157]
17	Midwest	Cumari – GO	1029	2.7	C	Borges et al. [33]
18	South	Moreira Sales – PR	42	4.8	C	Barbosa and Pavanelli [20]
19	South	Maringa – PR	150	16.0	C	Colli et al. [61]
20	South	Campo Mourao – PR	5219	7.2	C	Mortean et al. [144]
21	South	Maria Helena – PR	431	6.5	C	Santos and Merlini [177]
22	South	Cascavel – PR	343	17.8	C	Takizawa et al. [207]
23	South	Ubirata – PR	86	4.6	C	Falavigna et al. [79]
24	South	Campo Mourao – PR	86	4.6	C	Kulik et al. [117]
25	South	Jataizinho – PR	264	26.9	C	Lopes et al. [122]
26	South	Pitanga – PR	181	20.9	C	Nascimento and Moitinho [149]
27	South	Maringa – PR	369	5.9	C	Guilherme et al. [101]
28	South	Porto Alegre – PR	17,951	15.1	C	De Carli et al. [69]
29	South	Pelotas – RS	73	35.6	C	Jeske et al. [111]
30	South	Ipe – RS	124	4.0	C	Zanotto et al. [218]
31	South	Palmeiras das Missoes – RS	209	20.6	C	Nagel et al. [148]
32	South	Caxias do Sul – RS	257	1.5	C	Camello et al. [44]
33	South	Caxias do Sul – RS	331	3.3	C	Porto et al. [162]
34	South	Flores da Cunha – RS	341	3.2	C	Cavagnolli et al. [53]
35	South	Rio Grande – RS	144	28.5	C	Mata-Santos et al. [136]
36	South	Porto Alegre – RS	146	10.3	C	Silva et al. [192]
37	South	Caxias do Sul – RS	9787	14.6	C	Basso et al. [22]
38	South	Porto Alegre – RS	181	14.9	C	Bencke et al. [24]
39	South	Campos Novos – SC	109	13.7	C	Biolchi et al. [28]
40	South	Florianopolis – SC	3126	3.5	C	Bueno et al. [40]
41	South	Florianopolis – SC	57	31.6	C	Santos et al. [180]
42	South	Blumenau – SC	53	18.9	C	Andrade et al. [11]
43	South	Criciuma – SC	94	56.4	E	Schnack et al. [185]
44	South	Florianopolis – SC	43	4.6	C	Korzeniowski et al. [116]
45	Northeast	Teresina – PI	39,539	8.4	C	Ibiapina et al. [108]
46	Northeast	Burti dos Lopes – PI	511	8.4	C	Sousa et al. [201]
47	Northeast	Parnaiba – PI	251	29.9	C	Fernandes et al. [85]
48	Northeast	Sao Raimundo Nonato – PI	265	42.6	C	Alves et al. [10]
49	Northeast	Santa Cruz – RN	3480	2.3	C	Lima et al. [121]
50	Northeast	Aracaju – SE	476	31.3	C	Oliveira et al. [155]
51	Northeast	Aracaju – SE	500	32.6	C	Rollemberg et al. [172]
52	Northeast	Aracaju – SE	298	14.1	C and E	Lawson et al. [119]
53	Northeast	Santo Antonio de Jesus – BA	144	45.8	C	Reis et al. [167]
54	Northeast	Salvador – BA	48,028	0.5	C and M	Soares et al. [200]
55	Northeast	Santo Antonio de Jesus – BA	144	45.8	C	Andrade et al. [12]
56	Northeast	Aiquara – BA	236	15.7	C	Santos et al. [183]
57	Northeast	Feira de Santana – BA	349	50.1	C	Almeida et al. [9]
58	Northeast	Ilheus – BA	97	49.5	C and E	Santos et al. [181]
59	Northeast	Salvador – BA	200	65.0	C	Seixas et al. [186]
60	Northeast	Salvador – BA	52,704	3.4	C and M	Santos et al. [178]
61	Northeast	Salvador – BA	5624	15.6	C	Santos et al. [176]
62	Northeast	Ipira – BA	410	12.2	C	Santos-Junior et al. [184]
63	Northeast	Cuite – PB	45	40.0	C	Bezerra et al. [27]
64	Northeast	Joao Pessoa – PB	150	18.6	C	Monteiro et al. [143]
65	Northeast	Campina Grande – PB	1195	69.0	C and E	Silva et al. [195]
66	Northeast	Joao Pessoa – PB	67	28.3	C	Magalhães et al. [129]
67	Northeast	Campina Grande – PB	742	93.1	C	Silva et al. [188]
68	Northeast	Russas – CE	213	21.6	C and M	Calegar et al. [43]
69	Northeast	Fortaleza – CE	582	29.4	C	Bachur et al. [15]
70	Northeast	Fortaleza – CE	735	38.3	C and E	Braga et al. [36]
71	Northeast	Fortaleza – CE	161	20.5	E	Braga et al. [35]
72	Northeast	Fortaleza – CE	564	36.2	C and E	Braga et al. [34]
73	Northeast	Maceio – AL	1003	6.4	C and M	Santos et al. [182]
74	Northeast	Maceio – AL	1798	3.8	C and E	Duarte et al. [74]
75	Northeast	Recife – PE	213	4.7	C and E	Dourado et al. [72]
76	Northeast	Recife e Macaparana – PE	1783	5.8	C and M	Pinheiro et al. [159]
77	Northeast	Macaparana – PE	1437	2.6	C and M	Pinheiro et al. [158]
78	Northeast	Recife, Palmares e Bodoco – PE	633	28.3	C, Z and E	Aca et al. [3]
79	Northeast	Sao Lourenço da Mata – PE	485	41.2	C and E	Gonçalves et al. [98]
80	Northeast	Recife – PE	459	50.9	E	Okazaki et al. [153]
81	Northeast	Chapadinha – MA	3933	26.9	C	Silva et al. [190]
82	Northeast, North	Timo – MA, Macapa – AP	10,260	3.8	C	Ferraz et al. [86]
83	North	Belem – PA	320	3.7	C	Carvalho et al. [50]
84	North	Santarem – PA	367	34.3	C	Banhos et al. [16]
85	North	Belem – PA	334	28.4	C and E	Silva et al. [187]
86	North	Belem – PA	438	28.9	E	Póvoa et al. [163]
87	North	PA	300	57.6	C	Miranda et al. [140]
88	North	Presidente Figueiredo – AM	143	4.2	C	Gonçalves et al. [99]
89	North	Coari – AM	65	9.2	C	Silva et al. [194]
90	North	Santa Izabel do Rio Negro – AM	463	25.3	C	Valverde et al. [215]
91	North	Manaus – AM	400	40.5	C	Oliveira et al. [154]
92	North	Iauarete – AM	333	31.2	C	Boia et al. [32]
93	North	Manaus – AM	451	23.9	C	Maia et al. [130]
94	North	Coari – AM	211	29.4	C	Monteiro et al. [142]
95	North	Coari – AM	123	21.1	C	Silva et al. [189]
96	North	Sao Gabriel da Cachoeira – AM	895	29.9	C	Rios et al. [170]
97	North	Santa Izabel do Rio Negro – AM	308	71.7	C	Boia et al. [31]
98	North	Eirunepe – AM	413	38.2	C	Araújo and Fernandez [13]
99	North	Manaus – AM	1585	37.3	C and E	Benetton et al. [25]
100	North	Nova Olinda do Norte – AM	81	23.4	C	Hurtado-Guerrero et al. [106]
101	North	Novo Airao – AM	316	29.1	C	Boia et al. [30]
102	North	Manaus – AM	110	9.1	C	Giugliano et al. [96]
103	North	Ariquemes e Monte Negro – RO	216	50.4	C and E	Santos et al. [179]
104	North	Acrelandia – AC	429	25.6	C	Souza et al. [202]
105	Southeast	Diamantina – MG	66	18.2	C	Eustachio et al. [78]
106	Southeast	Belo Horizonte – MG	6289	6.5	C and M	Costa et al. [62]
107	Southeast	Viçosa – MG	419	32.9	C	Iasbik et al. [107]
108	Southeast	Alfenas – MG	277	2.5	C	Felizardo et al. [83]
109	Southeast	Ituiutaba – MG	140	22.1	C	Moura et al. [146]
110	Southeast	Sete Lagoas – MG	26	30.8	C	Pires et al. [160]
111	Southeast	Uberaba – MG	1323	6.4	C	Cabrine-Santos et al. [42]
112	Southeast	Caldas – MG	60	66.6	…	Simões et al. [199]
113	Southeast	Divinopolis – MG	1403	5.7	C and E	Pereira et al. [156]
114	Southeast	MG	409	89.7	C	Assis et al. [14]
115	Southeast	Uberaba – MG	82	63.4	M	Cembranelli et al. [54]
116	Southeast	Ouro verde de minas – MG	315	28.2	C	Carvalho et al. [49]
117	Southeast	Uberlandia – MG	110	17.3	C	Ferreira-Filho et al. [89]
118	Southeast	Viçosa – MG	246	4.1	C	Einloft et al. [75]
119	Southeast	Pato de Minas – MG	161	16.1	C	Silva and Silva [191]
120	Southeast	Berilo – MG	149	24.8	C	Martins et al. [135]
121	Southeast	Vespasiano – MG	176	16.5	C	Barçante et al. [21]
122	Southeast	Uberlandia – MG	160	23.1	C	Machado et al. [127]
123	Southeast	Abadia dos Dourados – MG	376	20.5	C	Machado et al. [128]
124	Southeast	Belo Horizonte – MG	472	14.6	C	Menezes et al. [138]
125	Southeast	Vespasiano – MG	537	6.3	C	Santos et al. [175]
126	Southeast	Bambui – MG	2811	7.4	C	Rocha et al. [171]
127	Southeast	Uberlandia – MG	264	1.5	C	Rezende et al. [169]
128	Southeast	Uberlandia – MG	104	24.0	C	Costa-Cruz et al. [63]
129	Southeast	Uberlandia – MG	100	62.0	C	Favoreto Jr and Machado [82]
130	Southeast	Sao Mateus – ES	50	36.0	C	Albuquerque and Souza [6]
131	Southeast	Sao Matheus – ES	42	19.0	C	Brauer et al. [39]
132	Southeast	Sao Mateus – ES	221	31.2	C	Damázio et al. [67]
133	Southeast	Sao Mateus – ES	82	31.7	C	Damázio et al. [66]
134	Southeast	Sumidouro – RJ	294	12.9	C	Barbosa et al. [19]
135	Southeast	Rio de Janeiro – RJ	3245	6.8	C	Faria et al. [81]
136	Southeast	Rio de Janeiro – RJ	595	12.2	C	Ignácio et al. [109]
137	Southeast	Rio de Janeiro – RJ	180	10.5	…	Valença-Barbosa et al. [214]
138	Southeast	Niteroi – RJ	68	17.6	C	Leite et al. [120]
139	Southeast	Niteroi – RJ	1749	5.4	C	Macedo et al. [126]
140	Southeast	Niteroi – RJ	429	11.6	C	Uchôa et al. [213]
141	Southeast	Rio de Janeiro – RJ	218	1.4	C	Carvalho-Costa et al. [51]
142	Southeast	Niteroi – RJ	140	15.7	C	Port-Lourenço et al. [161]
143	Southeast	Niteroi – RJ	261	21.8	C	Uchôa et al. [212]
144	Southeast	RJ	99	31.3	C	Moura et al. [145]
145	Southeast	Ribeirao Preto – SP	233	13.3	C	Fonseca et al. [91]
146	Southeast	Sao Jose do Rio Preto – SP	100	7.0	C	Castro et al. [52]
147	Southeast	Campos do Jordao – SP	185	22.2	C	Branco et al. [37]
148	Southeast	Mirassol – SP	310	15.1	C	Belloto et al. [23]
149	Southeast	Sao Jose do Rio Preto – SP	500	0.8	C	Cardoso et al. [48]
150	Southeast	Sao Paulo – SP	66	40.9	C	Lopes et al. [123]
151	Southeast	Catanduva – SP	133	9.7	C	Biscegli et al. [29]
152	Southeast	Presidente Bernardes – SP	101	8.9	C	Tashima et al. [209]
153	Southeast	Ribeirao Preto – SP	429	9.3	C	Capuano et al. [47]
154	Southeast	Araraquara – SP	503	14.5	C	Miné and Rosa [139]
155	Southeast	Sao Paulo – SP	120	16.6	C	Korkes et al. [115]
156	Southeast	Catanduva – SP	250	34.4	C	Faleiros et al. [80]
157	Southeast	Presidente Prudente – SP	1000	7.1	C	Tashima and Simões [208]
158	Southeast	Sao Paulo – SP	200	13.0	C	Cimerman et al. [58]
159	Southeast	Sao Jose da Bela Vista – SP	1032	0.2	C	Tavares-Dias and Grandini [210]
160	Southeast	Botucatu – SP	147	22.4	C	Guimarães and Sogayar [102]
161	Southeast	Holambra – SP	222	15.7	C	Kobayashi et al. [114]
162	Southeast	Sao Paulo – SP	407	1.5	C	Ferreira et al. [88]
163	Southeast	Osasco – SP	155	21.3	Z	Aca et al. [2]
164	Southeast	Sao Paulo – SP	395	25.8	C	Guerra et al. [100]
165	Southeast	Guarulhos – SP	913	21.9	C	Chieffi et al. [56]
166	Southeast	Ribeirao Preto – SP	1351	23.1	C	Ferriolli-Filho [90]
Animal host						
167	Southeast	Rio de Janeiro – RJ	13 (bird – emu)	23.1	C and M	Gallo et al. [93]
168	Southeast	Rio de Janeiro – RJ	1190 (non-human primate)	33.4	C	Barbosa et al. [18]
169	Southeast	Petropolis – RJ	790 (pig)	21.5	C	Barbosa et al. [17]
170	Southeast	Sao Paulo – SP	21 (rodent – mouse)	9.5	C	Chagas et al. [55]
171	Southeast	Bauru – SP	47 (non-human primate)	23.4	C	David et al. [68]
172	Southeast	Botucatu – SP	207 (bird)	1.9	C	Marietto-Gonçalves et al. [132]
173	Southeast	Sao Paulo – SP	31 (canid – guara wolf)	22.6	C	Gilioli and Silva [95]
174	Southeast	Sao Paulo – SP	103 (edentate – anteater)	4.8	C	Diniz et al. [71]
175	Northeast	CE – MA – PI – PE – BA	340 (dog)	3.8	C	Zanetti et al. [217]
176	Northeast	Aracaju – SE	44 (rodent – mouse)	2.3	C	Guimarães et al. [103]
177	Northeast	Lajes – RN	64 (sheep)	17.2	C	Souza et al. [203]
178	Northeast	Itabuna – BA	119 (dog)	0.8	C	Campos-Filho et al. [45]
179	Northeast	Recife – PE	685 (bird)	5.7	C	Freitas et al. [92]
180	North	Sena Madureira – AC	18 (bird)	22.2	C	Souza et al. [204]
181	Midwest	Caceres – MT	120 (dog)	15.8	C	Rosales and Malheiros [173]
182	South	SC	217 (goat)	1.8	C	Radavelli et al. [164]

*Abbreviations*: MT – Mato Grosso; PR – Parana; PI – Piaui; RN – Rio Grande do Norte; PA – Para; MG – Minas Gerais; SE – Sergipe; BA – Bahia; MS – Mato Grosso do Sul; ES – Espirito Santo; RJ – Rio de Janeiro; PB – Paraiba; RS – Rio Grande do Sul; SP – Sao Paulo; CE – Ceara; AL – Alagoas; SC – Santa Catarina; DF – Federal District (capital of Brazil); MA – Maranhao; AP – Amapa; AM – Amazonas; RO – Rondonia; GO – Goias; AC – Acre; PE – Pernambuco. C – conventional method, based on detection by optical microscopy; M – molecular method, based on DNA detection; E – Elisa method, serology-based; Z – zymodema method, based on isoenzyme analysis.

Regarding the methodological quality, according to the GRADE criteria used, all 166 studies evaluating the prevalence of *Entamoeba* spp. in different Brazilian populations as well as the 16 studies evaluating its prevalence in different animal host species presented a high methodological quality, all with a score of 5.

### *Entamoeba* spp. in the Brazilian population

Overall, the 166 studies on human samples included 268,465 coprological tests and 114 from the oral cavity, including samples from 24 Brazilian states and the Federal District. The only states not analyzed were Roraima and Tocantins, both in the northern region. Test distribution by state showed that 10 studies were performed in Bahia (representing 40.2% of the analyzed samples), 4 in Piaui (15.1%), 11 in Rio Grande do Sul (11.0%), 25 in Minas Gerais (6.1%), 10 in Parana (4.0%), 22 in Sao Paulo (3.3%), 11 in Rio de Janeiro (2.7%), 15 in Amazonas (2.2%), 6 in Pernambuco (1.9%), 6 in Santa Catarina (1.3%), 5 in Ceara (0.8%), 5 in Paraiba (0.8%), 5 in Para (0.6%), 7 in Mato Grosso do Sul (0.6%), 3 in Sergipe (0.5%), 8 in Mato Grosso (0.4%), and 4 in Espirito Santo (0.2%). Two studies were conducted in the states of Maranhao (1.6% of the included samples) and Alagoas (1.0%). Only one study was conducted in Amapa (3.7%), Rio Grande do Norte (1.3%), Goias (0.4%), Acre (0.2%), Rondonia (0.1%), and the Federal District (0.03%).

Of the 166 studies analyzed, only 19 distributed patient samples by sex, totaling 56,442 samples, of which 65% were female and 35% male, with 1992 (3.5%) positive samples. Of the positive samples, 1082 (54.3%) were from females and 910 (45.7%) from males.

Fifty-six studies distributed the samples by age group, totaling 35,411 samples. Of these samples, 26,143 (73.8%) were from children aged 0–9 years; 5971 (16.8%) from aged 10–19 years, and 3297 (9.4%) from adults aged over 19 years. Of these samples, 5684 (16.1%) were positive for *Entamoeba* spp., with 4133 (72.7%) from children aged 0–9 years, 609 (10.8%) from 10–19 years, and 942 (16.5%) from adults over 19 years.

Regarding the status of the immune system, 266,794 (99.3%) of the samples were from patients with no previously reported compromized immune system, whereas 1785 (0.7%) samples were from immunocompromized patients. Regarding the causes of immunosuppression, it was found that 1463 (82%) samples were from human immunodeficiency virus (HIV) carriers, 249 (14%) from patients undergoing hemodialysis, and 73 (4%) from patients with cancer. Of the samples from immunosuppressed patients, 338 (19%) were positive for *Entamoeba* spp.; 284 (84%) of these patients had HIV, 28 (8.3%) were undergoing hemodialysis, and 26 (7.7%) had cancer.

#### Pooled prevalence of *Entamoeba* spp.

**Figure 2:**
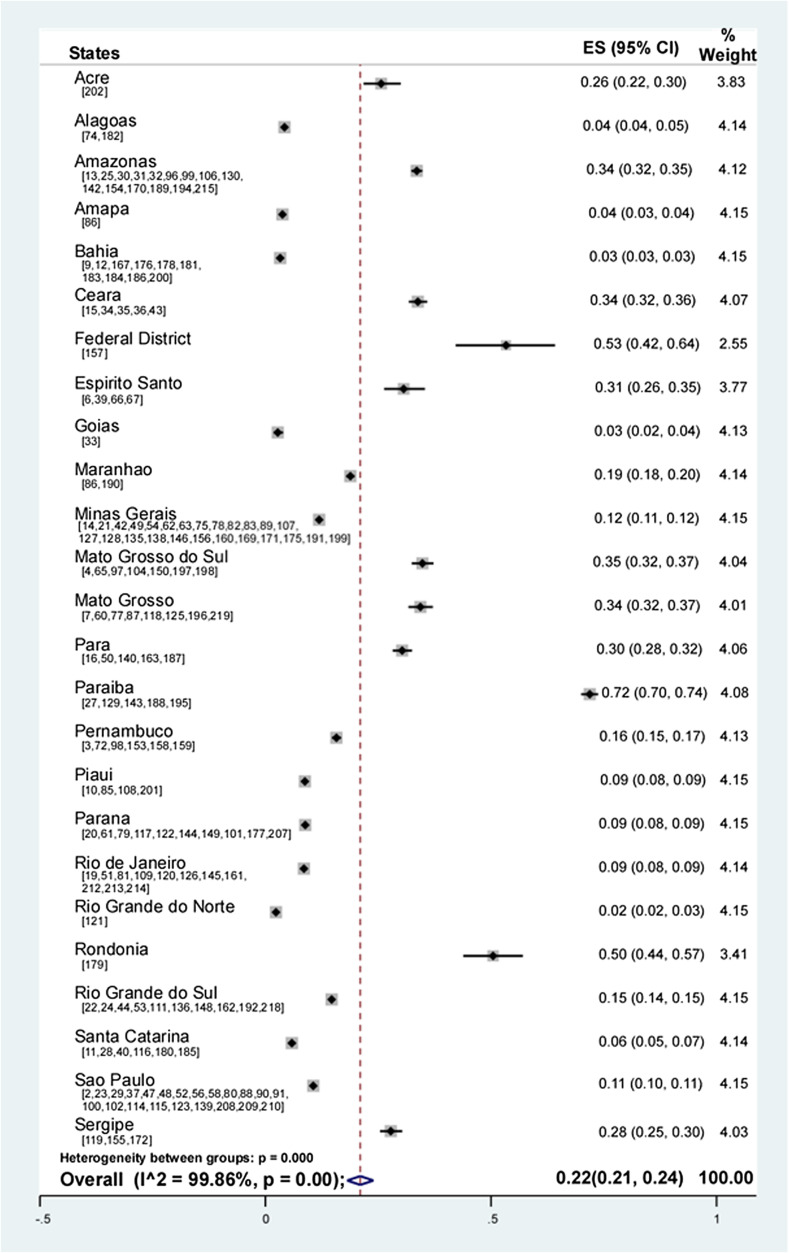
Forest plot for a random-effect meta-analysis of the pooled prevalence of *Entamoeba* spp. in the Brazilian population by state. In parentheses the studies used for each state.

The prevalence of *Entamoeba* spp. reported in the analyzed studies was between 0.2% and 93.1%. Random-effects meta-analysis showed a pooled prevalence of 22% (95% CI: 21–24; weight 100%) of *Entamoeba* spp. in the Brazilian population (Fig. 2).

The analysis of pooled prevalence by state showed that it was 72% in Paraiba, 53% in the Federal District, 50% in Rondonia, 35% in Mato Grosso do Sul, 34% in Mato Grosso and Amazonas and Ceara, 31% in Espirito Santo, 30% in Para, 28% in Sergipe, 26% in Acre, 19% in Maranhao, 16% in Pernambuco, 15% in Rio Grande do Sul, 12% in Minas Gerais, 11% in Sao Paulo, 9% in Parana, Piaui and Rio de Janeiro, 6% in Santa Catarina, 4% in Alagoas and Amapa, 3% in Bahia and Goias, and 2% in Rio Grande do Norte (Fig. 2). The pooled prevalence with complete 95% CI values for each state is shown in Table 2.

**Table 2:** Distribution of the pooled prevalence of *Entamoeba* spp. according to state and age.

Overall				≤9			10–19			>20		
State	Overall prevalence	95% CI	Weight (%)	Prevalence	95% CI	Weight (%)	Prevalence	95% CI	Weight (%)	Prevalence	95% CI	Weight (%)
PR	13	1–25	4.30	13	1–25	7.16	–	–	–	–	–	–
SE	31	27–36	1.44	31	27–36	2.39	–	–	–	–	–	–
RS	20	7–33	5.63	15	2–29	7.13	–	–	–	36	26–47	5.19
PA	34	30–39	1.43	34	30–39	2.38	–	–	–	–	–	–
MG	33	22–45	24.58	23	9–36	22.36	45	24–67	41.1	47	7–100	21.17
SP	19	13–26	12.89	17	10–24	14.31	34	28–41	10.49	21	19–23	10.72
MT	28	6–50	5.66	34	6–62	7.10	–	–	–	9	4–20	5.28
MA	4	3–6	1.45	4	3–6	2.41	–	–	–	–	–	–
AP	4	3–4	1.45	4	3–4	2.42	–	–	–	–	–	–
SC	36	13–58	4.06	36	13–58	6.79	–	–	–	–	–	–
PB	85	84–87	2.9	85	84–87	4.82	–	–	–	–	–	–
BA	30	17–42	6.3	13	9–16	4.18	50	28–72	6.99	20	16–25	10.50
AM	20	14–26	9.88	16	8–24	9.49	30	22–39	10.18	26	21–32	10.56
MS	56	36–76	5.50	55	45–64	2.29	75	65–83	10.11	51	44–57	10.48
RJ	22	17–27	2.74	21	16–27	2.38	–	–	–	26	15–40	5.12
PE	23	8–39	5.7	25	20–30	2.39	6	5–7	21.13	35	28–41	5.31
ES	19	10–33	1.33	–	–	–	–	–	–	19	10–33	5.16
FD	53	42–64	1.34	–	–	–	–	–	–	53	42–64	5.18
PI	30	25–36	1.42	–	–	–	–	–	–	30	25–36	5.33
Overall Prevalence	29	24–34	100	25	18–31	100	40	29–50	100	34	20–47	100

*Abbreviations*: 95% CI, 95% confidence interval. PR – Parana, SE – Sergipe, RS – Rio Grande do Sul, PA – Para, MG – Minas Gerais, SP – Sao Paulo, MT – Mato Grosso, MA – Maranhao, AP – Amapa, SC – Santa Catarina, PB – Paraiba, BA – Bahia, AM – Amazonas, MS, Mato Grosso do Sul, RJ – Rio de Janeiro, PE – Pernambuco, ES – Espirito Santo, DF – Federal District, PI – Piaui.

Pooled prevalence by age group showed that the age group between 10 and 19 years had the highest prevalence (40%; 95% CI: 29–50; weight 100%). The state with the highest prevalence in this age group was Mato Grosso do Sul (75%), followed by Bahia (50%), Minas Gerais (45%), Sao Paulo (34%), Amazonas (30%), and Pernambuco (6%). In the group over 19 years of age, the pooled prevalence was 34% (95% CI: 20–47; weight 100%). The state with the highest prevalence in this age group was the Federal District (53%), followed by Mato Grosso do Sul (51%), Minas Gerais (47%), Rio Grande do Sul (36%), Pernambuco (35%), Piaui (30%), Rio de Janeiro and Amazonas (26%), Sao Paulo (21%), Bahia (20%), Espirito Santo (19%), and Mato Grosso (9%). Children below 9 years of age had a pooled prevalence of 25% (95% CI: 18–31; weight 100%). The state with the highest prevalence for this age group was Paraiba (85%), followed by Mato Grosso do Sul (55%), Santa Catarina (36%), Mato Grosso and Para (34%), Sergipe (31%), Pernambuco (25%), Minas Gerais (23%), Rio de Janeiro (21%), Sao Paulo (17%), Amazonas (16%), Rio Grande do Sul (15%), Parana and Bahia (13%), and Maranhao and Amapa (4%) (Table 2).

The pooled prevalence in the 19,721 male samples was 26% (95% CI: 20–31; weight 100%). The state with the highest prevalence was Para (57%), followed by Pernambuco (33%), Amazonas (28%), Parana (20%), Espirito Santo (19%), Sao Paulo (18%), Mato Grosso and Rio de Janeiro (15%), Minas Gerais (8%), Mato Grosso do Sul (7%), and Bahia (1%). In contrast, the pooled prevalence in the 36,721 female samples was 29% (95% CI: 14–43; weight 100%). The state with the highest prevalence of *Entamoeba* spp. in female samples was Mato Grosso do Sul (62%), followed by Para (59%), Amazonas (33%), Espirito Santo (31%), Pernambuco (25%), Parana (21%), Sao Paulo (19%), Rio de Janeiro (11%), Minas Gerais (7%), and Mato Grosso (4%).

The pooled prevalence in immunosuppressed patients was 18% (95% CI: 7–30; weight 100%). The most prevalent cause of immunosuppression with *Entamoeba* spp. was cancer (36%), followed by HIV infection (27%), and hemodialysis (10%) (Table 3).

**Table 3:** Distribution of the pooled prevalence of *Entamoeba* spp. according to the type of immunosuppression.

Immunosuppression	Overall subtotal	95% CI	Weight (%)
Cancer	36	26–47	10.45
HIV infection	27	9–45	55.96
Hemodialysis	10	2–18	33.59
Overall prevalence	18	7–30	100

*Abbreviations*: 95% CI, 95% confidence interval.

### *Entamoeba* spp. in animals in Brazil

The 16 studies that analyzed the prevalence of *Entamoeba* spp. in animals included 3805 coprological tests in different species (79.1% mammals and 20.9% birds). The classification by direct interaction with humans showed that 54% were wild animals in captivity, 2.3% were free-living wild animals, 15.2% were pets, and 28.5% were farm animals.

**Figure 3:**
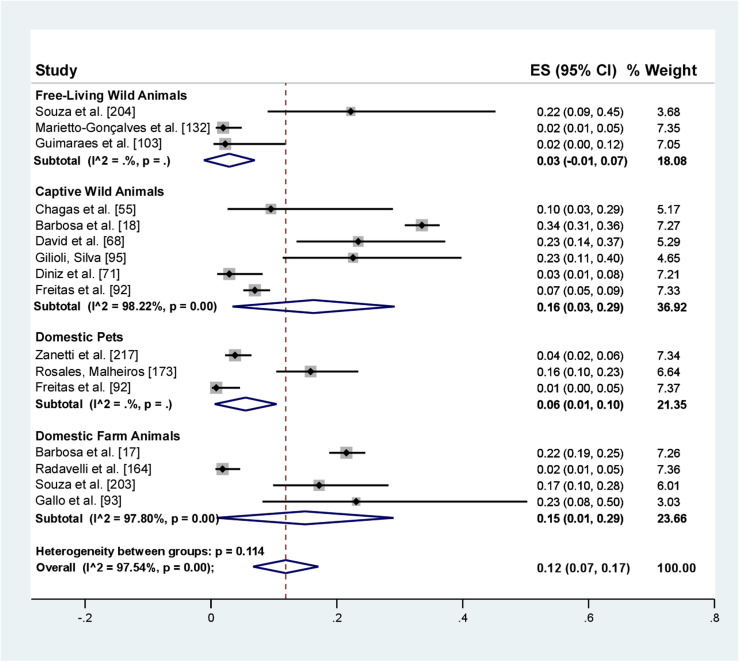
Forest plot for a random-effect meta-analysis of the pooled prevalence of *Entamoeba* spp. in different animals in Brazil, according to the type of interaction with humans.

The analysis of prevalence of *Entamoeba* spp. in Brazilian animals from different orders and with different types of human interaction showed a pooled prevalence of 12% (95% CI: 7–17). Wild animals in captivity had a prevalence of 16% (95% CI: 3–29), free-living wild animals 3% (95% CI: 1–7), farm animals 15% (CI95%: 1–29.00), and pets 6% (95% CI: 1–10) (Fig. 3).

The prevalence of *Entamoeba* spp. by taxonomic class showed a prevalence of 12% (95% CI: 6–19) in mammals and 6% (95% CI: 1–12) in birds (Table 4).

**Table 4:** Distribution of the pooled prevalence of *Entamoeba* spp. according to taxonomic class and interaction with humans.

Study	Taxonomic class	Overall prevalence (%)	95% CI	Weight (%)
	Mammals	12	6–19	78.60
Guimarães et al. [103]	Rodents	2	0–12	7.05
Chagas et al. [55]	Rodents	10	3–29	5.17
Barbosa et al. [18]	Non-human primates	34	31–36	7.27
David et al. [68]	Non-human primates	23	14–37	5.29
Gilioli and Silva [95]	Guara wolf	23	11–40	4.65
Diniz et al. [71]	Anteaters	3	1–8	7.21
Zanetti et al. [217]	Dogs	4	2–6	7.34
Rosales and Malheiros [173]	Dogs	16	10–23	6.64
Campos-Filho et al. [45]	Dogs	1	0–5	7.37
Barbosa et al. [17]	Pigs	22	19–25	7.26
Radavelli et al. [164]	Goat	2	1–5	7.36
Souza et al. [203]	Sheep	17	10–28	6.01
	Birds	6	1–12	21.40
Souza et al. [204]	Birds	22	9–45	3.68
Marietto-Gonçalves et al. [132]	Birds	2	1–5	7.35
Freitas et al. [92]	Birds	7	5–9	7.33
Gallo et al. [93]	Emus	23	8–50	3.03
	Interaction with humans			
	Free-living wild animals	3	1–7	18.08
	Captive wild animals	16	3–29	36.92
	Domestic pets	6	1–10	21.35
	Domestic farm animals	15	1–29	23.66

*Abbreviations*: 95% CI, 95% confidence interval

Of the captive wild mammals, non-human primates were the most studied, with prevalence percentages of 34% and 23%*.* In contrast, of the farm mammals, pigs had a prevalence of 22%. Notably, the only animal considered a pet in the studies analyzed was the dog, representing 16% (Table 4). Of the domestic farm birds, emus had a prevalence of 23% and free-living wild birds had a prevalence of 22% (Table 4).

### *Entamoeba* spp. diversity in different host species in Brazil

Conventional microscopy analysis, molecular characterization, serology, and isoenzyme analysis were used to identify *Entamoeba* spp. in 150 studies, totaling 17,651 human samples. In contrast, only six studies on host animals characterized 51 positive samples at the species level.

**Figure 4:**
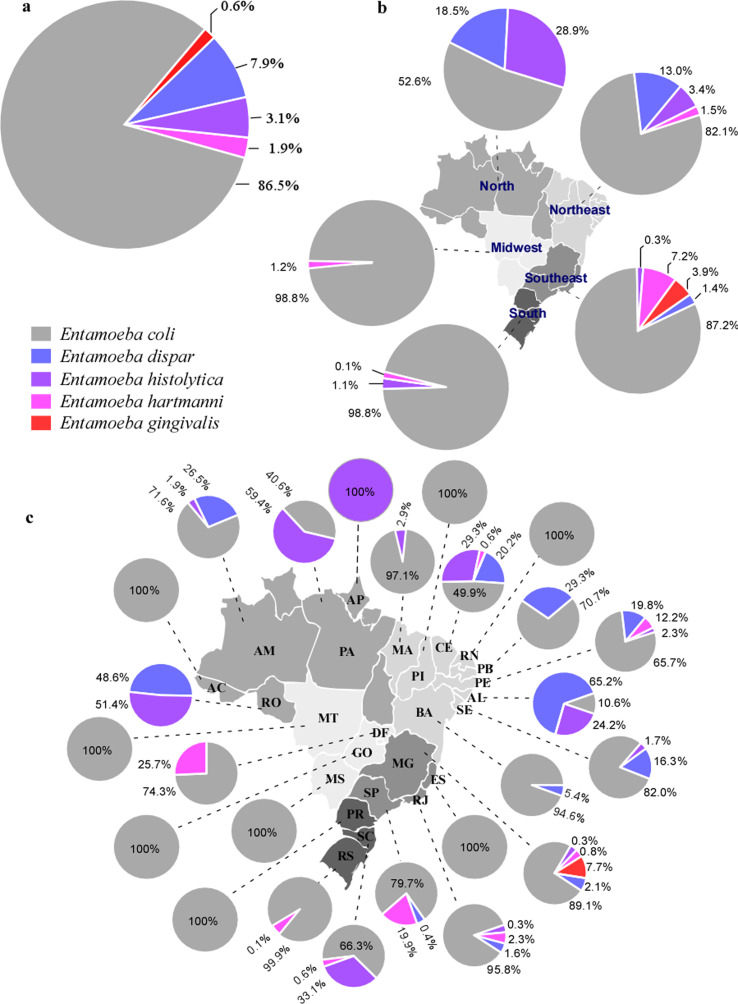
Geographical distribution of *Entamoeba* spp. detected in Brazil. (a) Species detected in 17,651 human samples. (b) Species distribution in human and animal hosts according to Brazilian regions. (c) Species distribution in human and animal hosts in Brazilian states. Abbreviations: AC – Acre; AM – Amazonas; RO – Rondonia; PA – Para; MA – Maranhao; PI – Piaui; CE – Ceara; RN – Rio Grande do Norte; PB – Paraiba; PE – Pernambuco; AL – Alagoas; SE – Sergipe; BA – Bahia; MG – Minas Gerais; ES – Espirito Santo; RJ – Rio de Janeiro; SP – Sao Paulo; PR – Parana; SC – Santa Catarina, RS – Rio Grande do Sul; MS – Mato Grosso do Sul; GO – Goias; MT – Mato Grosso; DF – Federal District (Capital of Brazil).

To calculate the prevalence of the reported species, only the samples that performed this procedure were used. For this purpose, 17,651 samples (fecal and oral cavity) with identification of *Entamoeba* species, were used. In these samples, the most prevalent species identified in human hosts were *E. coli* (86.5%), followed by *E*. *dispar* (7.9%), *E. histolytica* (3.1%), *E*. *hartmanni* (1.9%), and *E. gingivalis* 0.6% (Fig. 4). The species identified as non-pathogenic *E. histolytica*, through zymodeme [2, 3], were considered as *E. dispar*. On the other hand, *E. coli* was the only species with a taxonomic classification, identified in animal hosts. In addition, unidentified *Entamoeba* species were reported in animal hosts.

The prevalence of species by geographical regions showed that *E. coli* was the most prevalent species in the five regions, with high percentages. *Entamoeba histolytica* was identified in the north (28.9%), northeast (3.4%), south (1.1%), and southeast (0.3%) regions. The southeast region presented the greatest species diversity, with the identification of the five *Entamoeba* spp. registered in Brazil, followed by the northeast region with four species, north and south with three, and center-west with two different species (Fig. 4).

The detailed distribution of protozoan species by the Brazilian state is shown in Figure 4.

## Discussion

Data on the prevalence of *Entamoeba* spp. were documented in 24 of 26 Brazilian states and in the Federal District. In this meta-analysis, a pooled prevalence of 22% of *Entamoeba* spp. was found in the Brazilian population. The pooled prevalence was calculated with samples of studies published between 1962 to 2020, so this percentage represents an overall prevalence of *Entamoeba* spp. in different hosts during this period of time, in Brazil. These results reflect a sampling of the five Brazilian regions, but the northeastern, southern, and southeastern regions are better characterized since these regions present higher scientific production. The northeastern region contributed 38 articles, representing 63.3% of the samples analyzed in this meta-analysis, the southern region 27 studies (16.3%), the southeastern region 62 (12.3%), the northern region 23 (6.7%), and the central-western region 17 studies (1.4%).

The analysis of the prevalence of *Entamoeba* spp. by region showed contrasting realities within the states of each region. The northeastern region showed high pooled prevalence percentages in the states of Paraiba (72%), Ceara (34%), Sergipe (28%), Pernambuco (16%), Piaui (9%) and Bahia (3%). Alagoas and the Rio Grande do Norte showed another reality, with a prevalence of 4% and 2%, respectively. The central-western region showed high pooled prevalence in the Federal District (53%) and the states of Mato Grosso do Sul (35%) and Mato Grosso (34%), but the state of Goias presented a pooled prevalence of 3%. In the northern region, the states of Rondonia (50%), Para (30%), Acre (26%), Amazonas (30%) and Maranhao (19%) showed high percentages of prevalence, while and Amapa showed a prevalence of 4%. In the southeastern region, the states of Espirito Santo, Minas Gerais and Sao Paulo showed a pooled prevalences of 31%, 12% and 11% respectively, while Rio Janeiro presented a moderate prevalence of 9%. The same data were found for the southern region, where the state of Rio Grande do Sul had a high pooled prevalence of 15% and the states Parana and Santa Catarina had a moderate prevalence of 9% and 6%, respectively.

The differences in the prevalence of intestinal parasites among the Brazilian regions were recently documented in a previous study [81]. However, in addition to the differences among the regions, this present study showed great prevalence differences within the same region. This epidemiological data can be used as a tool to identify areas of social vulnerability as intestinal parasitosis is strongly associated with the socioeconomic level of the population. In contrast, Brazil is an extensive country and presents many regional and intraregional socioeconomic and health development differences. Only 39% of the cities collect and treat 100% of the sewage [38], with the lack of adequate basic sanitation system increasing the continuous dissemination of neglected diseases linked to sanitary problems, such as intestinal parasitosis, including those caused by *Entamoeba* spp.

Regarding sex, both showed a similar pooled prevalence of *Entamoeba* spp., with 29% for women and 26% for men, suggesting that sex may not be a determinant for protozoan contamination. Regarding age, there was a high prevalence in the three groups, 40% in the 10–19 years group, 34% in adults aged over 19 years, and 25% in children aged below 9 years.

Age is an important risk factor for intestinal parasitic infections. Children are often more susceptible to intestinal infectious diseases than adults owing to inadequate hygiene habits. Children aged below 9 years were the group that presented the highest number of samples analyzed in this meta-analysis, and even though it is the least prevalent for *Entamoeba* spp., 25% is a percentage of great importance within this population. In contrast, this study showed that the most prevalent group for *Entamoeba* spp. were the people aged 10–19 years. Therefore, school age represents a higher risk for amebiasis than the age of the general population. A previous study in Indonesia showed a high rate of *Entamoeba* spp. (52.8%) in the school-age (7–15 years) group [137]. The age group between 10 and 19 years was the most heterogeneous, including pre-adolescents, adolescents, and young adults. However, this group provides a possible panorama for the prevalence of intestinal parasitosis in high school students in Brazil.

The pooled prevalence of *Entamoeba* spp. infection in immunocompromized patients was 18%. This parasitic infection was most prevalent in cancer patients, with 36%, although they presented fewer samples for analysis, followed by HIV and hemodialysis patients, with a prevalence of 27% and 10%, respectively. Some studies indicate that this parasite frequently causes opportunistic infections in immunosuppressed patients [46, 111]; it was one of the most common causes of morbidity in this group. This study recorded high prevalence percentages in immunosuppressed patients, especially with cancer. Cancer, HIV, and hemodialysis patients become immunocompromized as a result of the disease itself or due to therapeutic procedures that cause immunosuppression [134, 193]. Although intestinal parasitic infections are a great risk with persistent diarrhea and severe clinical symptoms in immunocompromized patients, the routine diagnosis of these infections is often ignored during chemotherapy or disease [1, 131]. For this reason, it is extremely important to diagnose and treat parasitic infections to decrease morbidity in this group.

The overall pooled prevalence of *Entamoeba* spp. in animal hosts was 12%. Of these animals, *Entamoeba* spp. was most prevalent in mammals (12%), followed by birds (6%). Regarding human interaction, *Entamoeba* spp. was most prevalent in captive wild animals, which are not easily accessible to the general population, followed by domestic farm animals. Farm animal breeding is a possible risk factor for *Entamoeba* spp. transmission. Therefore, it is necessary to establish control measures to minimize the transmission of these parasites among different animal hosts and humans.

For *Entamoeba* spp. diversity, this study showed little variability in human hosts, with differentiation into five different species. Studies on animal hosts characterized only *E. coli*. Of the species identified in humans, *E. coli* was the most prevalent (86.5%), followed by *E. dispar* (7.9%), *E. histolytica* (3.1%), *E. hartmanni* (1.9%), and *E. gingivalis* (0.6%). The prevalence of these species in Brazil determined in this meta-analysis differed from the world scenario, which presented *E. dispar* with the highest prevalence (49.4%), followed by *E. histolytica* (32.3%), *E*. *coli* (1.9%), and *E. hartmanni* (0.9%) [64]. The Brazilian situation could be different if the 89 studies that used conventional identification methods also used molecular analysis in the 5234 samples to separate the species *E. dispar* from *E. histolytica*, which are morphologically indistinguishable and were not included in the general percentage.

Although this study presents the commensal parasite *E. coli* as the most prevalent in Brazil, it is important to highlight that this species has the same transmission route as that of other pathogenic species, such as *E. histolytica*, *E. dispar*, and even *Giardia lamblia* as well as helminths. The prevalence of this parasite can be used as an indicator of fecal/oral transmission, suggesting intestinal parasite transmission through water supply for human consumption or through contaminated food.

*Entamoeba histolytica* causes severe intestinal and extraintestinal amebiasis, representing a health risk in countries with inadequate sanitary barriers. This study identified significant prevalence and distribution percentages of *E. histolytica* in Brazil, with 28.9% prevalence in the north, 3.4% in the northeast, 1.1% in the south, and 0.3% in the southeast. In the central-western region, no study distinguished *E. histolytica* from *E. dispar*. It is important to note that more studies need to be developed in this region to resolve this sampling bias.

This study has some limitations. First, in human studies, some authors did not distribute the positive sample results by sex and/or age, decreasing the number of classified samples to better evaluate the prevalence by these variables. Second, many samples were not identified at the protozoan species level, which could improve data on the species distribution and prevalence in Brazil, especially those of the pathogenic *E. histolytica*. Finally, it is recommended that publication biases be evaluated using statistical methods in meta-analyses. However, the currently available methods, such as funnel graphs and the Egger regression test, are not considered useful in proportion studies [147].

In conclusion, this study showed a high prevalence of *Entamoeba* spp. in the Brazilian population (22%), with a prevalence of up to 50% in the northern, northeastern, and central-western regions. Although there were contrasting prevalence percentages among the regions, there is a wide distribution of *Entamoeba* spp*.* in Brazil. There was no difference between males and females, and the age group of 10–19 years had the highest prevalence, broadly indicating the prevalence of intestinal parasitosis in high-school students in Brazil. The most diagnosed species was *E. coli*, which may suggest the transmission of intestinal parasites through water supply for human consumption or through contaminated food. This may lead to the possibility of infection due to other protozoan pathogenic species. The pathogenic species *E. histolytica* is distributed in most Brazilian regions, with significant prevalence percentages. The prevalence in mammals was the highest among animals, with interactions among humans and captive, wild, or domestic farm animals presenting the higher protozoan prevalence.

The implementation of molecular methods to detect *Entamoeba* spp. in scientific productions is extremely important to reduce possible false-negatives using coprological methods and to differentiate protozoan species. Patients with any type of immunosuppression should undergo routine intestinal protozoa screening and early treatment to avoid future complications because a significant prevalence was identified in this population.

## Conflicts of interest

The authors declare that they have no conflicts of interest.
